# Supplementary material for: Illuminating understudied kinases: a generalizable biosensor development method applied to protein kinase N
Source: Commun Biol. 2025 Jan 22;8:109. doi: 10.1038/s42003-025-07510-4 (PMC11754634; doi:10.1038/s42003-025-07510-4)

Uncroped gels and corresponding autoradiograms

Gel image of autoradiogram used in Figure 2A

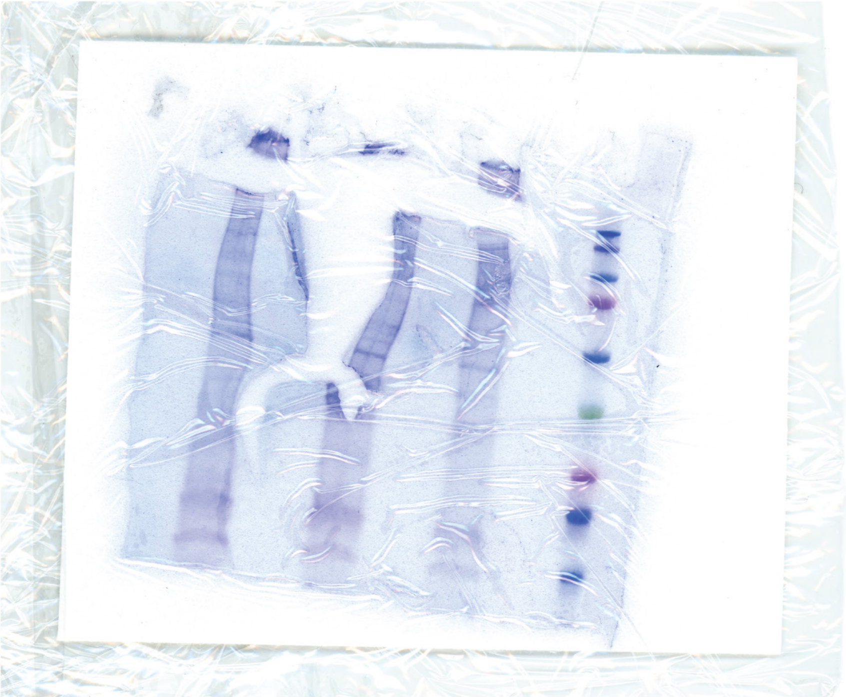

Autoradiogram used in Figure 2A

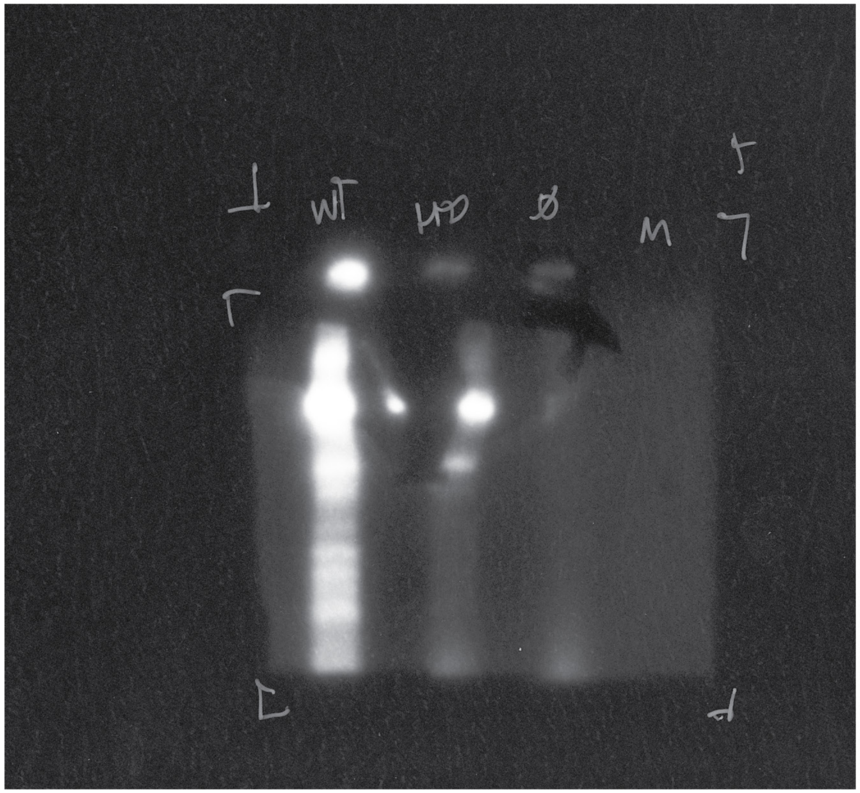

Gel image of autoradiogram used in Figure 3G

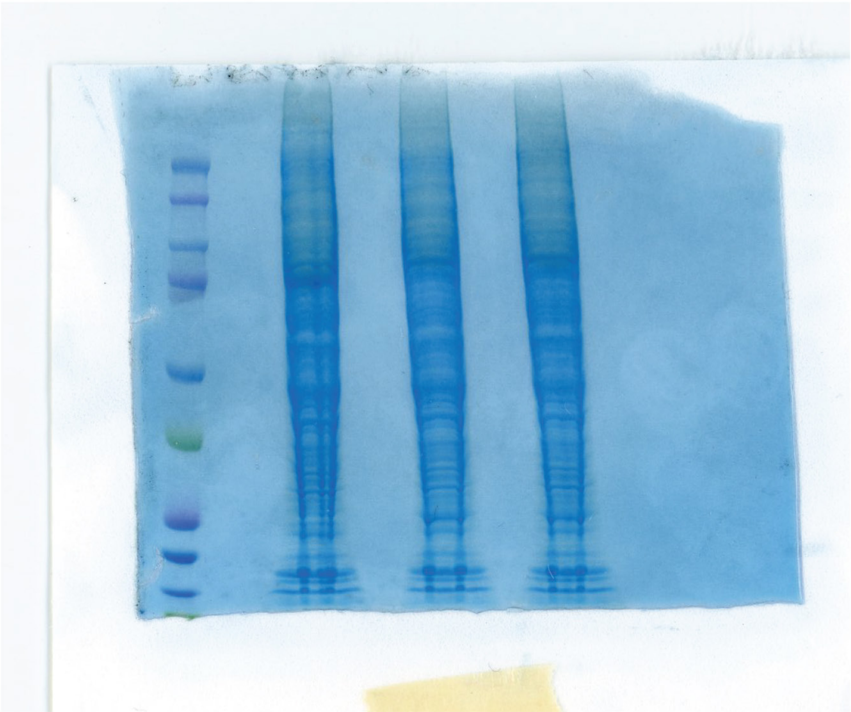

Autoradiogram used in Figure 3G

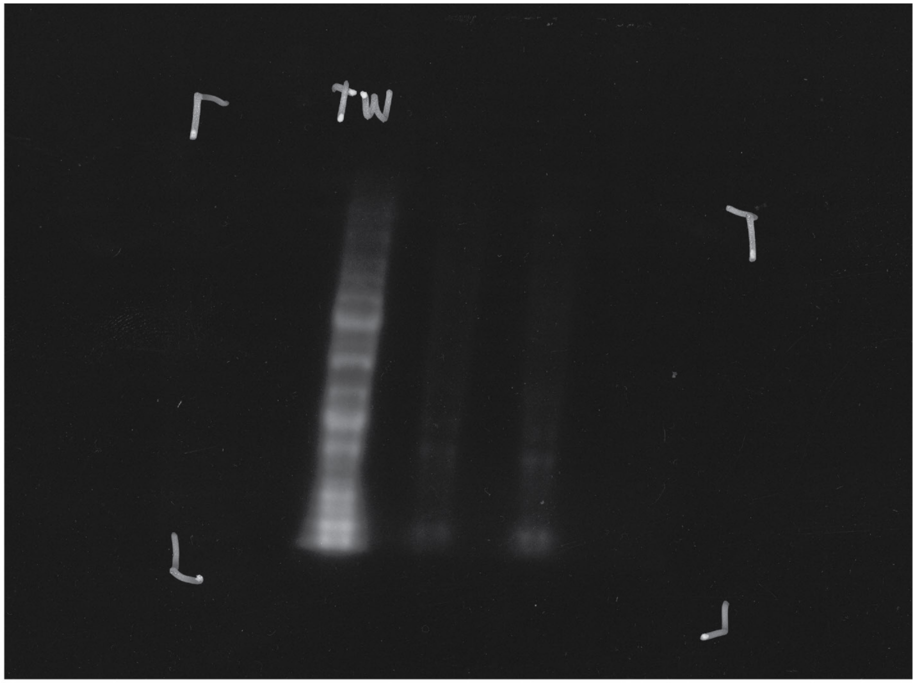

Supplement: Supplementary file 1 — Supplementary Information [file 42003_2025_7510_MOESM1_ESM.pdf]
