# Supplementary material for: Illuminating understudied kinases: a generalizable biosensor development method applied to protein kinase N
Source: Commun Biol. 2025 Jan 22;8:109. doi: 10.1038/s42003-025-07510-4 (PMC11754634; doi:10.1038/s42003-025-07510-4)
Supplement: Supplementary file 2 — Description of Additional Supplementary Materials [file 42003_2025_7510_MOESM2_ESM.pdf]

Description of Additional Supplementary Files

File name: Supplementary Data

Description: The raw data collected during this analysis, including kinase names, associated biosensors, and relevant publications
